# Molecular Cloning and Characterization of Violaxanthin De-Epoxidase (*CsVDE*) in Cucumber

**DOI:** 10.1371/journal.pone.0064383

**Published:** 2013-05-22

**Authors:** Xin Li, Wenchao Zhao, Xiyan Sun, Hongyu Huang, Lingcui Kong, Dandan Niu, Xiaolei Sui, Zhenxian Zhang

**Affiliations:** 1 Beijing Key Laboratory of Growth and Developmental Regulation for Protected Vegetable Crops, China Agricultural University, Beijing, China; 2 Ecological Laboratory, Ecotech Ecological Technology Ltd, Beijing, China; National Taiwan University, Taiwan

## Abstract

Violaxanthin de-epoxidase (VDE) plays an important role in protecting the photosynthetic apparatus from photo-damage by dissipating excessively absorbed light energy as heat, via the conversion of violaxanthin (V) to intermediate product antheraxanthin (A) and final product zeaxanthin (Z) under high light stress. We have cloned a violaxanthin de-epoxidase gene (*CsVDE*) from cucumber. The amino acid sequence of *CsVDE* has high homology with VDEs in other plants. RT-PCR analysis and histochemical staining show that *CsVDE* is expressed in all green tissues in cucumber and *Arabidopsis*. Using GFP fusion protein and immunogold labeling methods, we show that CsVDE is mainly localized in chloroplasts in cucumber. Under high light stress, relative expression of *CsVDE* and the de-epoxidation ratio (A+Z)/(V+A+Z) is increased rapidly, and abundance of the gold particles was also increased. Furthermore, *CsVDE* is quickly induced by cold and drought stress, reaching maximum levels at the 2^nd^ hour and the 9^th^ day, respectively. The ratio of (A+Z)/(V+A+Z) and non-photochemical quenching (NPQ) is reduced in transgenic *Arabidopsis* down-regulated by the antisense fragment of *CsVDE*, compared to wild type (WT) *Arabidopsis* under high light stress. This indicates decreased functionality of the xanthophyll cycle and increased sensitivity to photoinhibition of photosystem II (PSII) in transgenic *Arabidopsis* under high light stress.

## Introduction

Light is the ultimate source of energy for photosynthesis, but absorption of too much light that exceeds photosynthetic capacity is harmful to photosynthetic organisms. Plants and algae have evolved a series of mechanisms to protect themselves from photo-oxidative damage, such as chloroplast avoidance movement at the cellular level, photophobic movement as in *Chlamydomonas*, nonphotochemical quenching (NPQ), and redox-regulated changes in gene expression in response to excess light [1]. The xanthophyll cycle plays a central role in thermal dissipation of excessive light energy (a process known as NPQ). Zeaxanthin (Z), the product of the xanthophyll cycle, is formed by de-epoxidation of violaxanthin (V) via antheraxanthin (A), and accumulates in plants exposed to excess light [2], [3], [4], [5]. This process is catalyzed by violaxanthin de-epoxidase (VDE) in high light conditions. In low light conditions, Z is converted back to V by zeaxanthin epoxidase (ZEP) [6], [7], [8].

Under excessive light, a decrease in thylakoid lumen pH causes the soluble luminal protein VDE to attach to the thylakoid membrane where its substrate V is located. VDE is the first described putative plant lipocalin [9], and has been classified as a lipocalin-like protein [10]. The central lipocalin catalytic domain was proposed to be the binding site for the hydrophobic V substrate [11]. Furthermore, VDE uses ascorbate as a co-substrate, and is proposed to be monomeric. Its active site is occluded with a lipocalin barrel at neutral pH. An acidic luminal condition caused by high light, results in the opening of the barrel, and activation and dimerization of the enzymes [11].

VDE homologs have a conserved C-terminal tail that contains a high number of Glu residues and an N-terminal transit peptide that targets the protein to chloroplast [10], [11]. The regulation of VDE could take place at transcriptional, post-transcriptional or translational levels. It has been suggested that the VDE expression pattern is influenced by light and other environmental factors [12], [13], [14], [15], [16]. *VDE* genes have been isolated and purified from a number of species, but little is known about the relationship between localization, functionality and molecular mechanisms.

Cucumber is an important horticultural crop worldwide; it is prone to photoinhibition under high light stress at mid-day during the summer growing season. Many environmental stresses, such as drought and cold, can further limit the ability of cucumber to utilize light energy and photoinhibition under these circumstances can be increased. Expression analyses on wheat lipocalins and lipocalin-like proteins showed that low temperature induces the accumulation of *VDE* at the transcriptional level [10]. These studies support the idea that the xanthophyll cycle may scavenger potentially harmful molecules and thus protect the photosynthetic apparatus under abiotic stresses.

The main goals of this study are: 1) to isolate the cucumber *VDE* gene and its promoter in order to characterize its function and analyze its homology in plants and algae; 2) to locate the *CsVDE* at tissue and subcellular levels using histochemical staining, GFP fusion protein and immunogold labeling; 3) to study the expression of *CsVDE* under high light and other stress conditions; 4) to introduce *CsVDE* in the antisense direction in *Arabidopsis* for further studies of its role in the protection against excess light, and to understand the molecular mechanism of *CsVDE* and the xanthophyll cycle in response to excess light.

## Results

### Isolation, sequence analysis and expression of *CsVDE*

**Figure 1:**
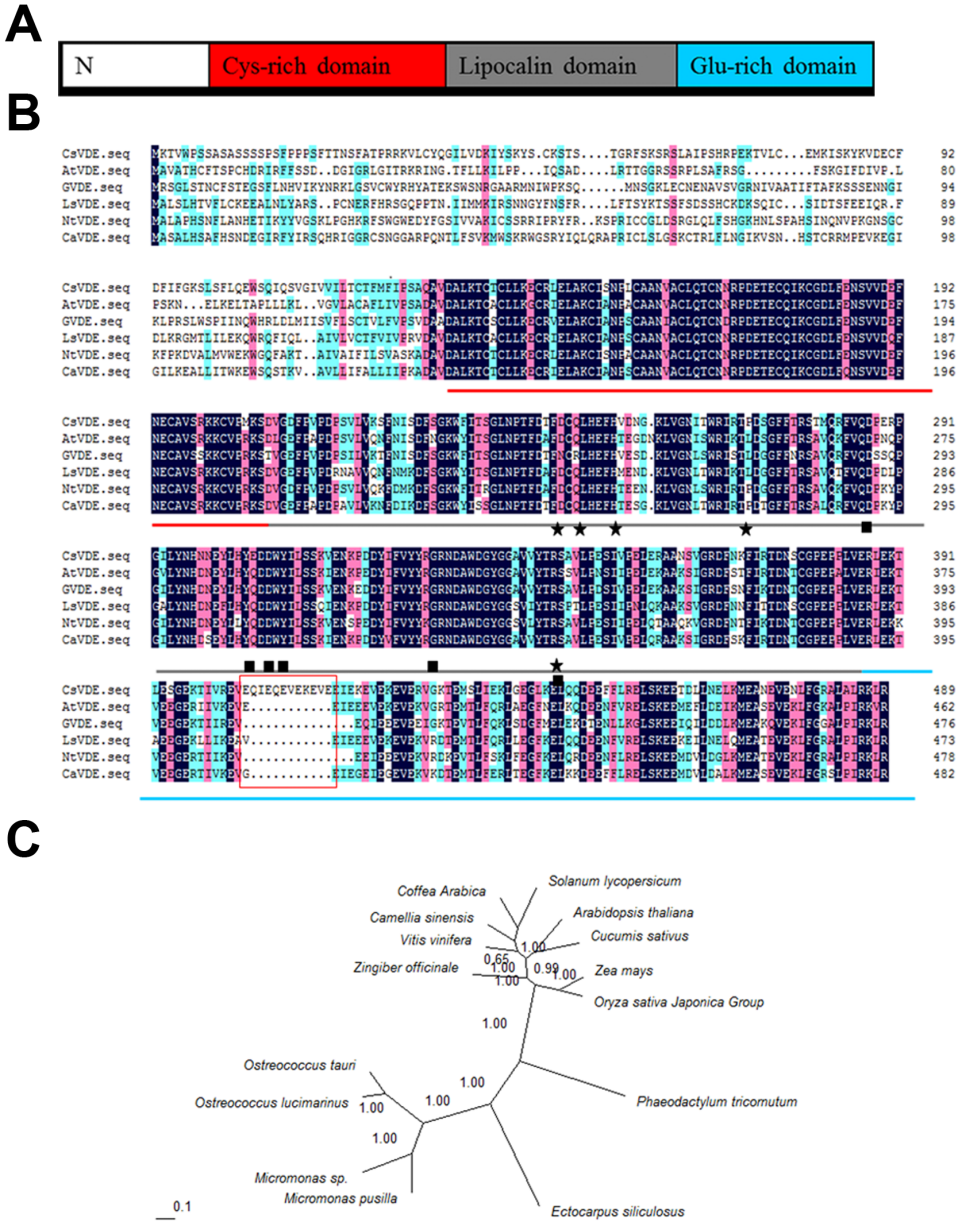
Amino acid sequence alignment and phylogenetic analysis of CsVDE and homologous proteins. (A) Schematic description of CsVDE domains. (B) Alignment of the deduced amino acid sequences of VDE in different plants. Red lines indicate the VDE Cys-rich domain; Gray lines indicate the Lipocalins domain; Blue lines indicate the Glu-rich domain. The important residues for pH switch are marked with black stars and the putative active site residues with black squares reference from Arnoux et al. (2009) [11]. *Arabidopsis thaliana* VDE (accession No. AEE28305), *Zingiber officinale* VDE (accession No. AAX59986), *Lactuca sativa* VDE (accession No. AAC49373), *Nicotiana tabacum* VDE (accession No. AAC50031), *Coffea arabica* VDE (accession No. ABB70816) sequences are shown. Black indicates 100% homology of the amino acid. Red indicates 75% homology of the amino acid. Green indicates 50% homology of the amino acid. (C) Phylogenetic analyses of selected VDEs. Phylogenetic studies were carried out using MrBayes3.1.2 and viewed with the TreeView package. All the trees were obtained with 200,000 generations for the chains, a sample frequency of a 10, and a burn in of 5,000 (ngen = 200000; Samplefreq = 10; burnin = 5,000). *Camellia sinensis* VDE (accession No. AAL67858), *Vitis vinifera* VDE (accession No. XP_002267152), *Osterococcus tauri* VDE (accession No. XP_003083515), *Ostreococcus lucimarinus* VDE (accession No. XP_001421704), *Micromonas sp.* VDE (accession No. XP_002503106), *Micromonas pusilla* VDE (accession No. XP_003061123), *Ectocarpus siliculosus* VDE (accession No. CBJ26509), *Phaeodactylum tricornutum* VDE (accession No. XP_002178643), *Oryza sativa* Japonica Group VDE (accession No. AAL83562), *Zea mays* VDE(accession No. NP_001147756), *Solanum lycopersicum* VDE (accession No. ACM92036).

A 1470 bp full length open reading frame (ORF), encoding 489 amino acids, of *CsVDE* was amplified from cucumber. The deduced amino acid sequence of *CsVDE* shares high homology with the VDEs in other plants, which includes a non-conserved N-terminal region and an approximate 150 amino acid downstream conserved VDE superfamily region containing a Cys-rich domain, a lipocalin domain, and a Glu-rich domain (Fig. 1A and B).

To investigate the genetic relationship of VDE genes among different species, phylogenetic analysis for homologs in algae and a few plant species was conducted (Fig. 1C). CsVDE is grouped into the plant VDEs clade and is more closely related to the VDE of Arabidopsis thaliana than to other plant species. VDEs from algae not only have distant relationships with those in higher plants, they also have less similarity with each other.

**Figure 2:**
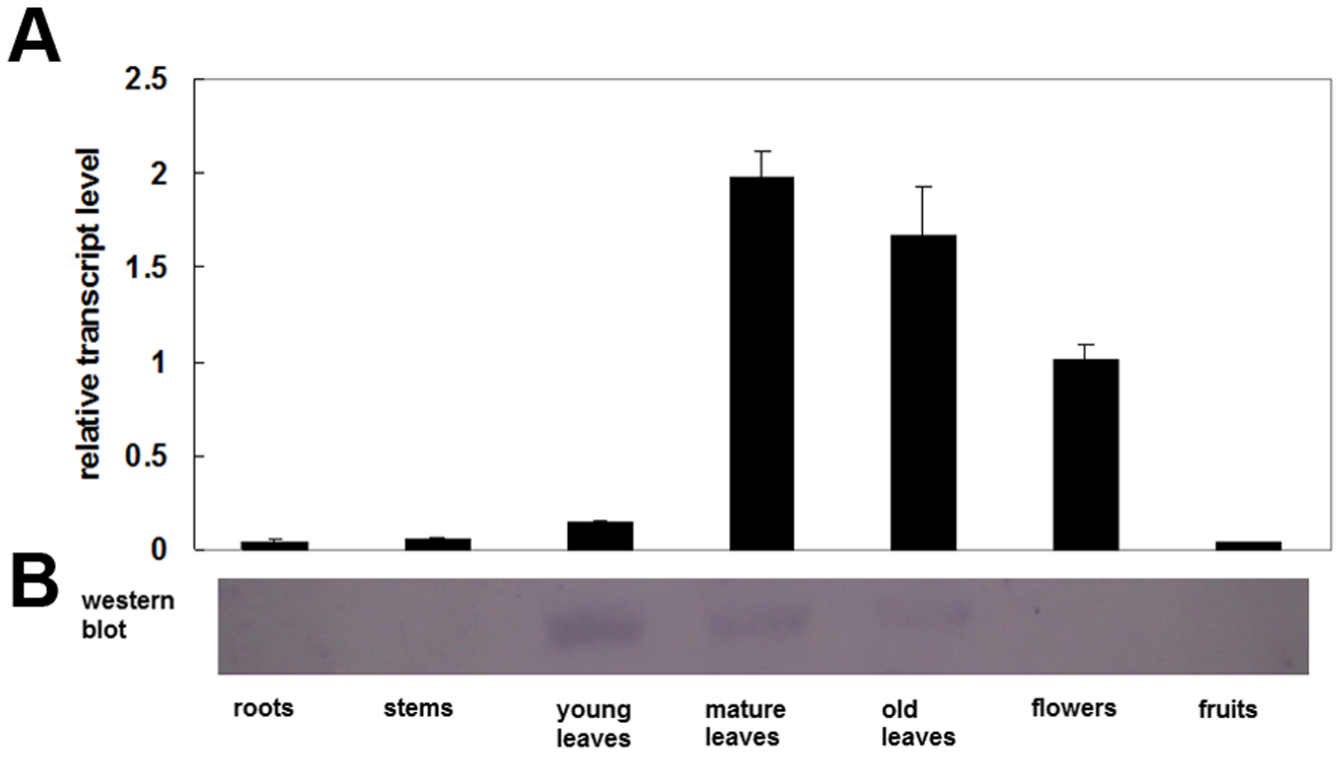
*CsVDE* transcript abundance (A) and Western blot analysis (B) in different plant tissues of cucumber. Plants were grown in the greenhouse until fruits appeared, and samples were taken from different tissues for quantitative real-time PCR and Western blot analysis. Each value is the mean ± standard deviation of three replicates.

Quantitative real-time PCR and Western blotting were used to determine the abundance of CsVDE at both the mRNA and protein levels. The spatiotemporal expression analysis showed that although present in all tissues examined, the transcripts of *CsVDE* were more abundant in mature leaves, old leaves, and flowers, but less abundant in fruits, roots, stems and young leaves (Fig. 2A).

Protein expression was only found in young leaves and mature leaves, but was not detected in old leaves and other tissues (Fig. 2B). However, the transcript level in young leaves was much lower than that in mature leaves, old leaves and flowers, suggesting that post-transcriptional or post-translational regulation were involved in various tissues.

### GUS activity analysis of the 2.0 kb *CsVDE* promoter in transgenic *Arabidopsis*

A 2 kb upstream fragment of the ATG codon of *CsVDE* was isolated from WT cucumber, and fused in front of GUS in PCAMBIA1391 vector. We introduced the construct into the *Arabidopsis* genome by *Agrobacterium*-mediated transformation. Several independent transgenic lines were identified and selected for histochemical analysis of promoter activity.

**Figure 3:**
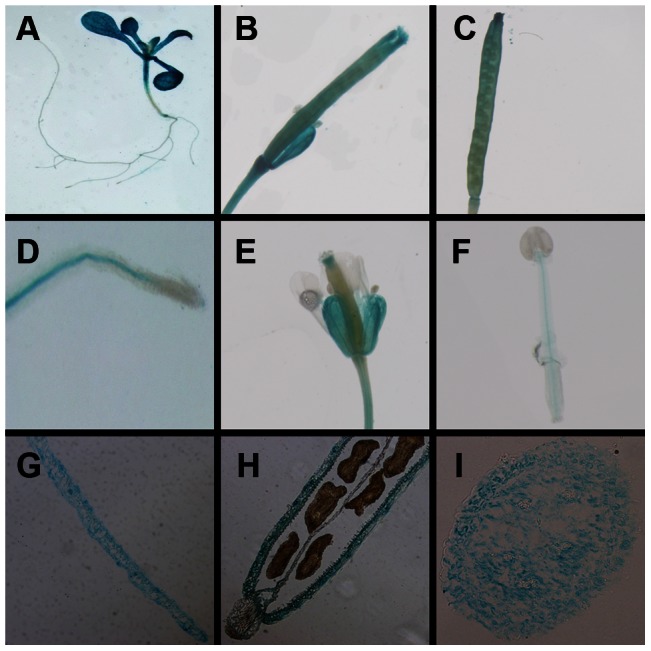
Histochemical analysis of *CsVDE* promoter regulated GUS expression in different tissues of transgenic *Arabidopsis*. (A) whole seedling, (B) ovary, (C) mature fruits, (D) root, (E) stem and flower, (F) stamen, paraffin sections of (G) a mature leaf, (H) mature fruit and (I) stem.

GUS activity was observed in the cotyledons, true leaves and hypocotyls of young seedlings of transgenic *Arabidopsis* plants (Fig. 3A). It could also be seen weakly in the stele of root, but could hardly be seen in the *root* apex (Fig. 3D). During the development of flowers and fruits, GUS expression was mainly localized in ovaries (Fig. 3B), mature fruits (Fig. 3C), floral stems, sepals (Fig. 3E), and vascular tissues of stamen (Fig. 3F). Only weak blue staining was observed on stigmas and filaments, and none on petals and anthers (Fig. 3E). Histochemical assays showed that strong GUS activity was localized to green organs, which correlated to the transcript profiling analysis in cucumber.

Paraffin sections of mature leaves, mature fruits and stems (Fig. 3G–I) show that GUS signal was observed in all parts of leaves and stems (Fig. 3G and I), but could only be seen in the epidermis of mature fruits, which was considered to be a green organ.

### Sub-cellular localization of *CsVDE*

**Figure 4:**
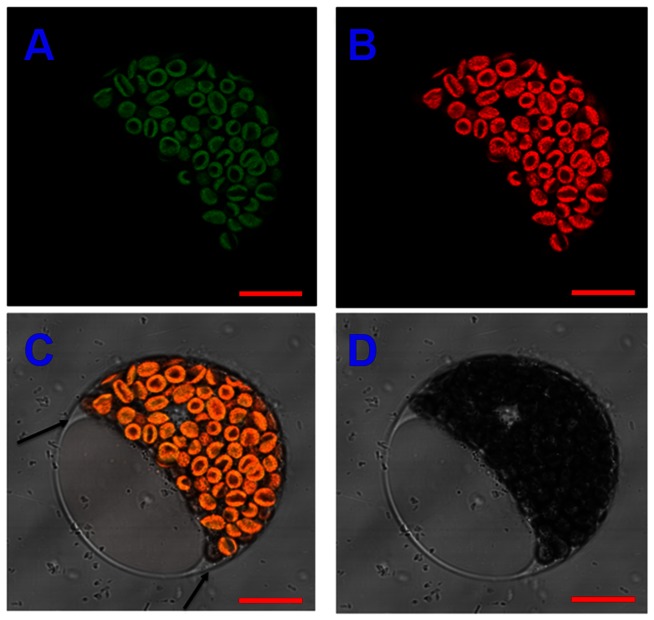
Subcellular localization of CsVDE in cucumber protoplast. (A) GFP (CsVDE-GFP fusion protein) fluorescence imaging, (B) chloroplast auto fluorescence imaging, (C) overlap image of GFP (green) and chlorophyll (red) fluorescence and (D) bright-field image. Bars = 10 µm.

Sub-cellular localization of *CsVDE* was determined by transiently expressing CsVDE using green fluorescent protein (GFP) fusion protein (CsVDE-GFP) in cucumber protoplasts. Results indicated that the fusion protein was target to the chloroplasts (Fig. 4). No GFP was found in the cytoplasm or cell membrane (as arrows show) of cucumber cotyledon protoplasts.

**Figure 5:**
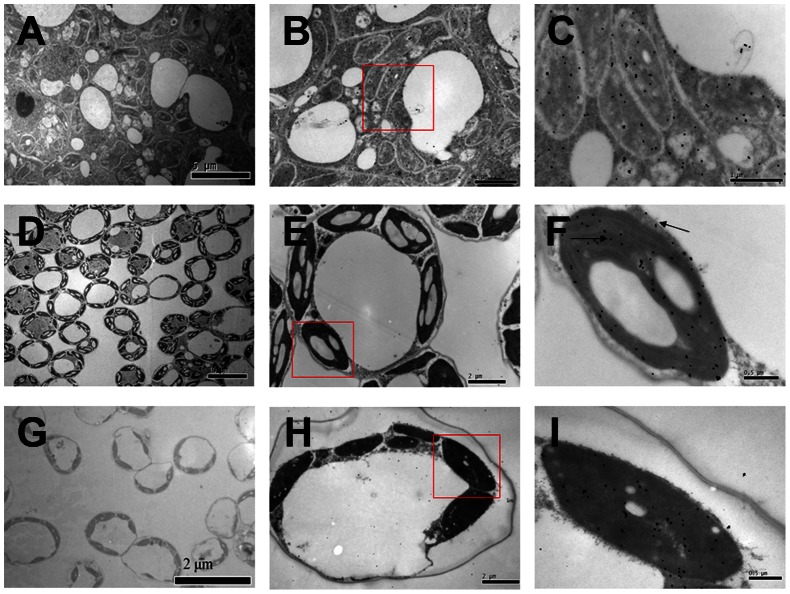
Immunogold localization of VDE in cucumber leaves. Samples were taken from young leaves (A.B.C), mature leaves (D.E.F) and old leaves (G.H.I). C, F and I are the close-up images of the boxed-in areas of B, E and H, respectively.

The localization of CsVDE in leaves was further studied through immunogold electron microscopy with CsVDE antibodies. We observed that the ultra-structure of cucumber leaf cells in different development stages was clearly different (Fig. 5). Young leaf cells did not have well developed organelles (Fig. 5A and B). Gold particles were found evenly distributed in the chloroplast and cytosol of these cells (Fig. 5B and C). Some particles were also found in an apparent vesicle part of vacuoles (Fig. 5C). Mature leaf cells had well developed and orderly arranged organelles and vacuoles (Fig. 5D and E). Most of the gold particles were distributed in chloroplasts and very few of them were in the cytosol (as shown by an arrow) of these leaf cells (Fig. 5E and F). Cells in the old leaf were undergoing apoptosis, and the organelles were losing part of their structures (Fig. 5G and H). We also observed plasmolysis in old leaf cells (Fig. 5H) and gold particles were mainly retained in chloroplasts (Fig. 5I).

The predominant chloroplast localization of CsVDE in mature and old leaves is consistent with its photo protective role under light stress (Fig. 5F and I).

### Effect of high light on *CsVDE*

**Figure 6:**
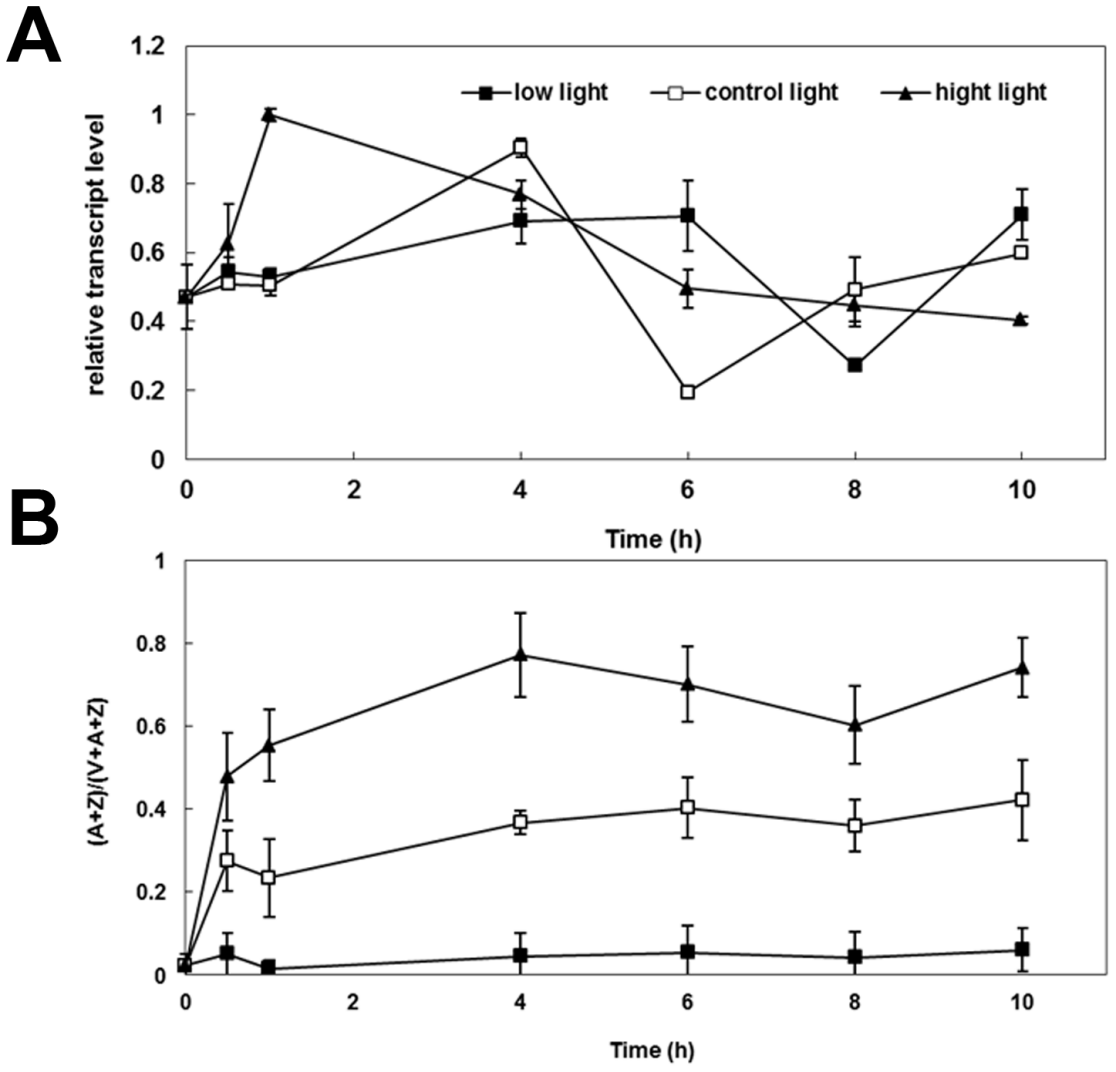
Relative transcript level and de-epoxidation ratio of CsVDE in WT cucumber under different light conditions. (A) Response of *CsVDE* to high light, control light and low light in wild-type cucumber leaves. High light: 1200 µmol m^−2^ s^−1^; control light: 500 µmol m^−2^ s^−1^ and low light: 100 µmol m^−2^ s^−1^, (B) De-epoxidation ratio (A+Z)/(V+A+Z) of WT cucumber under different light conditions.

In order to ascertain the effect of photon flux density on *VDE* gene expression, WT cucumber plants were exposed to 1200 µmol m^−2^ s^−1^ (high light) or 100 µmol m^−2^ s^−1^ (low light) for 10 h, respectively (with 500 µmol m^−2^ s^−1^ light condition as control light) (Fig. 6).

Under control light conditions, the transcript level of *CsVDE* gradually increased at the beginning of the day, peaked at 4 h and then gradually decreased to a minimum at 6 h, whereafter it increased to its original level (Fig. 6A).

Under low light conditions, the expression pattern of the *CsVDE* transcript was similar with that under control light conditions, but the peaking time of both the highest and the lowest levels were delayed by 2 h. Under high light conditions, *CsVDE* responded more rapidly and reached maximum expression in one hour, and then declined gradually until the 10^th^ hour without a recovery response. This lack of recovery phase is presumably due to the damages on the photosynthetic organs during a prolonged high light treatment.

Fig. 6B shows that the variation of xanthophyll cycle pigment pool ratio (A+Z)/(V+A+Z) in wild-type cucumber depended mainly on light intensity during treatment. De-epoxidation ratios of xanthophyll cycle pigments (A+Z)/(V+A+Z) in WT cucumbers were significantly and consistently increased under high light compared to either normal or low light, although the ratio fluctuated to some extent (Fig. 6B).

The de-epoxidation ratios of xanthophyll cycle pigments (A+Z)/(V+A+Z) in WT cucumbers were maintained at lower levels with little fluctuation during low light treatment. During normal light treatment, the ratio increased in the first half hour, after that, the ratios remained around 0.3. By contrast, high light promoted a rapid increase of the ratios, which were maintained at a higher level (between 0.6–0.73). Thus the variation and range of de-epoxidation ratios of xanthophyll cycle pigments of cucumber depends mainly on light intensity, especially under high light.

**Figure 7:**
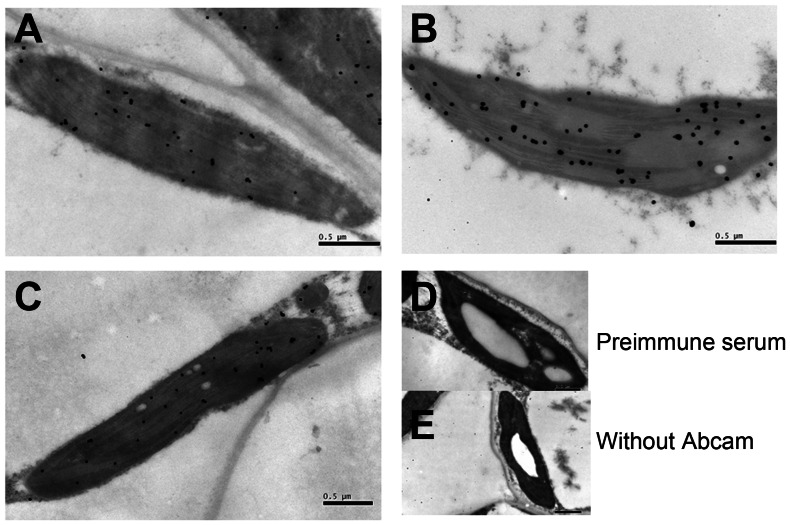
Immunogold localization of *CsVDE* in mature leaves of cucumber under different light treatments. (A) before light treatment (0 h), (B) high light (1200 µmol m^−2^ s^−1^) treatment for 10 h, (C) control light (500 µmol m^−2^ s^−1^) treatment for 10 h, (D) pre-immune serum control, (E) without Abcam control. Bars = 0.5 µm.

Fresh leaves exposed to different light intensities were used to determine protein location and gold particle density of CsVDE using immunogold labeling. The result showed that the gold particles were mainly distributed in the chloroplasts of all mature leaves. Under high light, at the 10^th^ h compared to 0 h and 10 h under control light treatment (Fig. 7 B, A and C), the majority of gold particles were observed in the lamella and the number of gold particles increased. No gold particles were detected in conditions without antiserum and with the pre-immune serum (Fig. 7D and E), confirming the specificity of antibody and reliability of the immunolocalization assay.

### Effects of drought and cold on *CsVDE*

**Figure 8:**
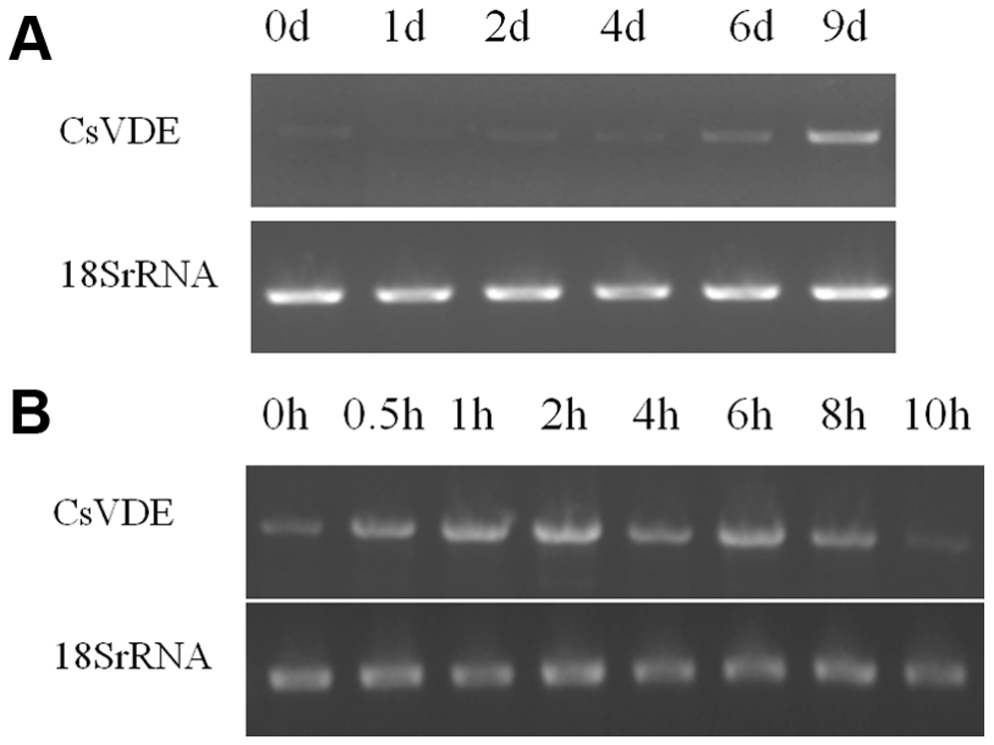
Effects of drought (A) and cold (B) stress on *CsVDE*. Total RNA was extracted from the wild type cucumber during drought and cold treatments. mRNA levels of *CsVDE* were examined by RT-PCR analysis, with 18s RT-PCR products as internal controls.

When plants were exposed to drought stress, the expression level of *CsVDE* gradually increased, reaching maximum on the 9^th^ day (Fig. 8A). To investigate whether low temperature has an effect on *CsVDE*, cucumber seedlings were treated under 4°C from 0 to 10 h. The results showed that low temperature stimulated *CsVDE* mRNA expression in the beginning, and reached maximum at 2 h after treatment, then gradually declined (Fig. 8B). This indicates that *CsVDE* expression is modulated by drought and cold stress.

### Antisense expression and functional analysis in transgenic *Arabidopsis* of *CsVDE*

Previous studies have indicated that *VDE* is a single copy gene in *Arabidopsis* [17], [18]. The down-regulation of *AtVDE* in transgenic *Arabidopsis* plants by the antisense *CsVDE* fragment was checked by RT-PCR. Many lines showed down regulation of 30 to 80%. None of the transgenic lines showed any visible phenotypic change (data not shown), although the NPQ at the physiological level was decreased under high light. This observation was the same as the *Arabidopsis npq1* mutant, in which the complete elimination of the function of VDE did not cause any morphological changes, but the NPQ level in the mutant was reduced under high light condition [17]. A transgenic line with 80% down regulation was chosen for further physiological analysis.

**Figure 9:**
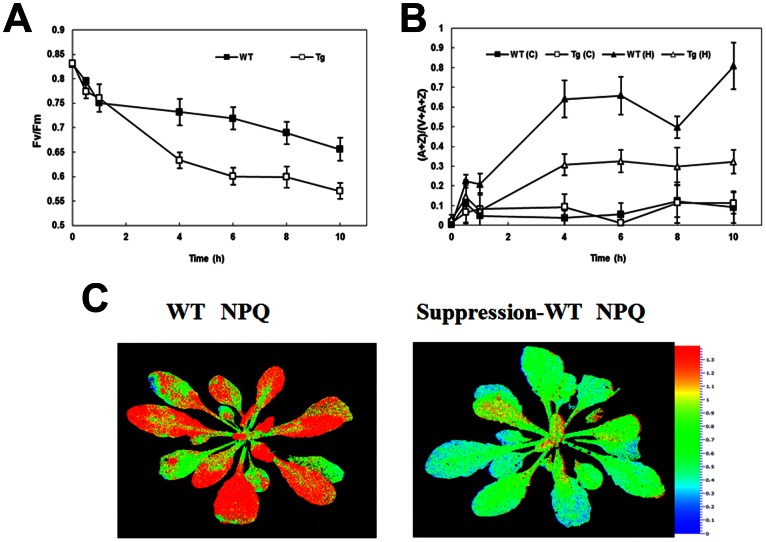
Different fluorescence parameters of wild-type (WT) and transgenic plants (Tg) under different light condition. (A) The effect of high light (1200 µmol m^−2^ s^−1^) stress on *F*v/*F*m of WT and Tg *Arabidopsis* (B) De-epoxidation ratio (A+Z)/(V+A+Z) of WT and Tg during high light (1200 µmol m^−2^ s^−1^) and control light (100 µmol m^−2^ s^−1^). (C) Screening for WT or Tg *Arabidopsis* by Video Imaging of Chlorophyll Fluorescence Quenching. Transgenic *Arabidopsis* and wild-type *Arabidopsis* were exposed to 500 µmol m^−2^ s^−1^ for 1 min and Chlorophyll fluorescence images of NPQ were generated using a Fluor Cam fluorometer.

Both WT and the transgenic antisense *Arabidopsis* plants were exposed to different light intensities to detect their responses to high light stress (Fig. 9). The *F*v/*F*m dropped sharply in the first hour in both lines. By the end of the 10 h treatment, The *F*v/*F*m in WT and transgenic decreased from 0.8 to 0.66 and 0.57, respectively, indicating that transgenic plants are somewhat more sensitive to high light damage (Fig. 9A).

The xanthophyll cycle pigment pool (A+Z)/(V+A+Z) in WT increased more than that in transgenic *Arabidopsis* leaves under different light treatments. But higher light intensity had a more profound effect during this process (Fig. 9B). At the end of 10 h high light stress, the (A+Z)/(V+A+Z) ratio in WT and transgenic lines reached 80% and 30% of their initial values, respectively, while no significant changes were detected during control light treatment (Fig. 9B).

Chlorophyll fluorescence image analysis showed that the NPQ of transgenic plant was significantly lower than that of WT when they were exposed to high light for a short period of time (Fig. 9C). This implied that more thermal energy transformed from excessive light energy was dissipated in the WT than in the transgenic line.

## Discussion

### Homology analysis

Amino acid sequence alignment of CsVDE showed high similarity at middle and C terminal regions of the protein with VDE proteins from other species. These regions contain the VDE superfamily domain, Cys-rich, lipocalin and Glu-rich domains. The lipocalin domain is thought to bind xanthophyll molecules in all-trans configuration, and has a series of conserved residues that are thought to be important for pH-mediated regulation and as the putative active site [11], [19].

Site directed mutagenesis and chemical modifications [11], [19] did suggest that a His residue plays a key role in pH-dependent conformation changes, since it ensures that the pKa values are ideal for pH sensor activity [11], [20], [21]. Some conserved residues in the active site have also been studied, such as a hydrogen bond-stabilized dimer in VDE, salt bridges, and several charged or polar residues (Fig. 1 black squares) [11]. *CsVDE* has all the key conserved residues, suggesting it has a common regulation of activity as other VDEs. Fig. 1 shows that the *CsVDE* gene has 11 additional residues in the C-terminal glutamic acid-rich domain, and five of them are Glu (Fig. 1 red box). The partial protonation of Glu residues at acidic pH (the optimal pH of active VDE) has been proposed to be important for the binding of VDE to the thylakoid membrane [22], [23]. We hypothesize that the additional 11 residues may enhance the binding capacity of CsVDE.

To explore the genetic relationship of CsVDE with VDEs from different species, a phylogenetic tree was generated. Our data suggests that plant VDEs have a distant genetic relationship with algal VDEs, in agreement with a previous phylogenic study [22]. The close phylogenetic relationship between *CsVDE* and *AtVDE*, the high sequence similarity, and the difficulty of cucumber transformation prompted us to use *Arabidopsis* as transgenic material for further functional analysis of CsVDE.

### Tissue specificity and subcellular localization

Similar to previous observation for *AtVDE*, *LeVDE* and *GVDE* [13], [14], [15], [16] expression, GUS signal and protein levels for *CsVDE* were most abundant in photosynthetic tissues,especially in mature leaves (Fig. 2 and 3). Furthermore, *CsVDE* expression was higher in flowers than in young leaves, similar to the observations with *AtVDE*. In agreement with previous results from *GVDE* [14], [16] and *LeVDE* [13], GUS activity driven by *CsVDE* promoter was also detected in sepals (Fig. 3), which may have caused the high expression of transcripts in flowers (Fig. 2). North et al. (2005) noted that *AtVDE* mRNA, but not the protein, was found in the root and concluded that the gene may be post-transcriptionally regulated [15]. The same situation was found in flowers, indicating likely post-transcriptional degradation of CsVDE proteins in non-photosynthetic tissues.

VDE from spinach [6] was found to be localized to thylakoids membrane vesicles. In this study, using GFP fusion protein and immunogold labeling, CsVDE proteins were mainly localized to chloroplasts in cucumber cotyledon protoplasts, mature leaf cells and old leaf cells.

It should be noted that transcription levels, protein levels and immunogold localization of CsVDE in young cucumber leaves do not correlate well with each other. It has been documented that the steady-state epoxidation status of the xanthophyll cycle (A+Z)/(V+A+Z) ratio in wild type *Arabidopsis* is roughly similar in young and mature leaves [24]. Despite a lower transcript level, the protein content of *CsVDE* in young leaves was the highest (Fig. 2), suggesting that *CsVDE* may be important to protect the photosynthetic apparatus from photo-damage in young leaves. Compared to mature leaf and old leaf cells, immunogold localization was not chloroplast specific in young leaf cells (Fig. 5). We speculate that there may be a post-translational regulatory mechanism at the N-terminal transit peptide of *CsVDE* in cytoplasm that targets the proteins to chloroplasts in mature and old leaf cells. This mechanism may be absent in immature cells in young leaves, leading to the lack of specific localization in the chloroplasts. Another hypothesis is that there may be some transport protein that interacts with VDE to transport this protein to chloroplasts. The expression level of this unknown transport protein may be lower in young leaves compared to mature leaves, thus VDE proteins are unable to be transported into chloroplasts in immature young leaf cells.

### Effects of high light and other stress factors on *CsVDE*

Light intensity changes shifted the pattern of the CsVDE expression. Under low light conditions, a delayed expression of VDE took place compared to that in normal light in cucumber, which shifted the maximum and minimum expression of the gene to later time points (Fig. 6).

The most important function of VDE is to respond to high light stress and protect plants from photo-damage. Previous studies have shown that high amount of VDE transcription in tobacco seedlings in response to short exposure to middle light intensity is decreased with prolonged illumination periods [25]. Our study showed that *CsVDE* transcription was quickly induced after 1 h of high light exposure, and declined thereafter. However, *AtVDE* gene transcription and GUS activity promoted by *GVDE* promoter have been shown to decrease under high light treatment [15], [16]. This conflict could be explained by a different sampling timing point in the two experiments. In the experiment done by North and Zhao, the sampling might not have been early enough to detect the quick response of the VDE gene to high light stress. The de-epoxidation ratio (A+Z)/(V+A+Z) became significantly higher under high light than under normal light, and no distinct changes were detected under low light, which is consistent with previous studies of VDE in other plants [24]. We observed an increased number of immunogold particles after high light treatment, indicating that VDE protein synthesis became activated under high light and may not be directly related to the expression level of *CsVDE* transcripts. This result is similar to those of previous observations [13], [15].

Compared to WT plants, *Arabidopsis* with antisense *CsVDE* transformation has decreased maximum efficiency of PSII photochemistry (*F*v/*F*m) (Fig. 9), indicating that transgenic plants are more sensitive to photo damage. One important protective mechanism of the xanthophyll cycle from excessive light is that the product (Z) of VDE has a thermal dissipation capacity, which is manifested by NPQ. The relationship between NPQ and de-epoxidation state has been previously discussed [26], [27], [28]. *Arabidopsis* with mutated *npq1* has been shown to be defective in the VDE and exhibit strongly inhibited NPQ [17]. In our experiment, the lower NPQ, (A+Z)/(V+A+Z) ratio and *F*v/*F*m in antisense plants indicated that they had a lower thermal dissipation capacity, fewer converted A and Z, and a more severe photo-inhibition under excessive light. These results suggest that the activity of VDE is partially suppressed in transgenic *Arabidopsis* and that VDE plays an important role in protecting plants from damage of excessive light. As discussed above, VDE is a single copy gene in *Arabidopsis*. The weaker suppression, compared to the *npq1* null mutant described by Niyogi et al. (1998) [17], of VDE activity in our transgenic plants probably is due to the less efficient down regulation of the gene via the antisense approach.

The effects of drought and cold stresses to the expression of *CsVDE* have also been observed. Drought stress increased *CsVDE* expression level (Fig. 8A). Low temperature initially enhanced *CsVDE* expression level (Fig. 8B), but it then declined; prolonged low temperature stress may result in physical damage. De-epoxidation of V has been shown to be increased by desiccation treatment in the absence of light in the foliose lichen *L. pulmonaria* [29]. Other studies have also shown that low temperature induces chronic photoinhibition of PSII, and enhances the de-epoxidation ratio of xanthophylls cycle pigments (A+Z)/(V+A+Z). Those results demonstrated a critical function of the xanthophyll cycle in protecting the photosynthetic apparatus from damage by light under low temperatures [13], [30], [31], [32]. Whether the induction of VDE caused by the different environmental stresses leads to decreased maximum photosynthetic capacity or increased sensitivity of PSII photo-inhibition is still unknown.

In conclusion, we have demonstrated that the physiological response of *CsVDE* to high light is related to VDE subcellular localization, but is not directly correlated to the expression of CsVDE under high light conditions. The expression of *CsVDE* is rapidly induced under different abiotic stress conditions, including drought, cold and hight light stress. Suppression of CsVDE increases the photo-inhibition of PSII by changing the level of de-epoxidation of the xanthophylls.

## Materials and Methods

### Plant material

Cucumber seeds ‘Deltastar’ (*Cucumis sativus* L.) were sown in 30×30 cm plastic pots containing peat: vermiculite (2∶1, v/v) in a phytotron. Plants were grown under 500 µmol m^−2^ s^−1^ light, at 25/18°C, in a 10 h light/14 h dark cycle. When plant had 4–5 true leaves, the third fully expanded leaf, counted from the shoot tip, was sampled from some plants. Other cucumber plants were grown in a greenhouse until fruits appeared, and samples were taken from different tissues for analysis. We define young leaves as the unexpanded ones on the shoot tip; mature leaves as the third fully expanded leaves counted from the tip; old leaves as the second leaves counted from the base of the stem. *Arabidopsis thaliana* (ecotype Columbia [Col-0]) plants were grown in our phytotron under 100 µmol m^−2^ s^−1^ light, at 22°C, on a 10/14 h day/night cycle.

### Cloning of the *CsVDE* gene and its promoter

Total RNA was extracted from 200 mg fresh cucumber leaves with Trizol reagent (Invitrogen, USA) and used as template for synthesis of single-strand cDNA using PowerScript™ Reverse Transcriptase. Genomic DNA was extracted from cucumber leaves using the cetyltrimethy ammonium bromide (CTAB) method [33]. The sequence of *CsVDE* was initially identified from the Cucumber Genomics database following homologous alignment of *VDE* genes from other species. Forward primer, 5′-TTATCTTAGTTTTCTAAGAGCAAGTGC-3′, and reverse primer, 5′-ATGAAAACCGTCTGGCCCTC-3′, were used to clone the gene from cucumber. The cloned gene was turned out to be the same as latterly deposited cucumber VDE gene with the accession number: HM590934.1. A 1983 bp upstream promoter region of the gene, with forward primer 5′-ACGCGTCGACTCTAGGATTAGTAGATCGATTGTTACC-3′, and reverse primer, 5′-CATGCCATGGGGCGCATCAGTGATTTGAAAAGAAG-3′, was also amplified. The accession for the promoter is JF719918.1.

### Vector construction and *Agrobacterium*-mediated transformation of *Arabidopsis*

The cloned cDNA of *CsVDE* was inserted in the antisense orientation into the binary vector pBI121 at *SacI* and *XbaI* sites. The *CsVDE* promoter was inserted into the binary vector PCAMBIA1391 between *SalI* and *NocI* site. The resulted plasmids, named PBI121-*CsVDE* and PCAMBIA1391-*CsVDE*P, respectively, were transformed into *Agrobacterium tumefaciens* EHA105. *Arabidopsis thaliana* plants (WT) were transformed by the floral dip method [34] using *Agrobacterium* strain EHA105. The T2 generation was used for physiological analysis.

### Protein extraction and Western blot

Total protein was extracted from cucumber roots, stems, flowers, old leaves, mature leaves, and young leaves with HEPES-NaOH buffered solution (50 mM HEPES-NaOH (pH 7.5), 2 mM MgCl_2_, 2 mM Na_2_-EDTA, 5 mM DTT). Protein concentrations were determined using bovine serum albumin as a standard.

Sodium dodecyl sulfate polyacrylamide gel electrophoresis (SDS-PAGE) and immunoblotting of the *CsVDE* were carried out according to Zhang et al. (2001) [35]. Briefly, after electrophoretic transfer from the polyacrylamide gels, the polyvinylidene fluoride membranes were blocked overnight at 4°C and incubated for 2 h at 37°C in the primary *CsVDE* antibody (The CsVDE poly-clonal antibody was generated by immunization of rabbits, and its specificity was confirmed by immunoblotting prepared by ABmart company http://www.abmart.cn/). Following extensive washes, the membranes were incubated with goat anti-rabbit IgG-alkaline phosphatase conjugate. The membranes were stained with 5-bromo-4-chloro-3-indolyl phosphate and nitro blue tetrazolium. The abundance of CsVDE proteins in different organs of wild type cucumbers was measured by Western blotting.

### High light, drought and low temperature treatments

When the cucumber seedlings had grown to a stage with 4 to 5 mature leaves, they were exposed to high light (1200 µmol m^−2^ s^−1^), low light (100 µmol m^−2^ s^−1^) or control light (500 µmol m^−2^ s^−1^) for 0 h, 0.5 h, 1 h, 4 h, 6 h, 8 h and 10 h. RNA was extracted from the leaves of cucumber growing under these different light conditions. For drought stress treatment, cucumber plants were grown to 4 leaves with regular watering, followed by the withdrawal of water for 9 days. Samples were taken at 0 d, 1 d, 2 d, 4 d, 6 d and 9 d. For low temperature treatment, cucumber plants were transferred to a growth chamber at 4°C. Mature leaves were sampled at 0 min, 30 min, 1 h, 2 h, 4 h, 6 h, 8 h, and 10 h. All the collected samples were snap frozen in liquid nitrogen before being stored at −80°C.

### Quantitative Real-Time PCR

*CsVDE* gene expression was carried out by real-time RT-PCR with the Real MasterMix (SYBR Green). Specific primers (5′-TCCTGGCATACTCTACAACCATAAC-3′ and 5′-CATACCCATCCCAAGCATCGT-3′) for *CsVDE* were used in real-time analysis. All quantifications were normalized to *α-Tubulin* cDNA fragments amplified in the same conditions by the following primers: 5′-ACGCTGTTGGTGGTGGTAC-3′ and 5′-GAGAGGGGTAAACAGTGAATC-3′ [36]. Real-time RT-PCR experiments were repeated three times, with the threshold cycles (C_T_) determined in triplicates. The average for the triplicate of one representative experiment was used in all subsequent analyses. The relative levels of *CsVDE* transcription were calculated using the 2^−ΔΔ*C*T^ method [37].

### Histochemical analysis of GUS activity and paraffin sections

Histochemical observations of leaves, roots, stems, flowers and cotyledons were performed by immersing and incubating the tissues in GUS-staining solution (1 mM 5-bromo-4-chloro-3-indoyl glucuronide (X-Gluc) in 100 mM phosphate buffer (pH 7.0), 10 mM Na_2_-EDTA, 1 mM potassium ferricyanide, 1 mM potassium ferrocyanide and 0.1% Triton X-100) at 37°C overnight. Stained samples were dehydrated in 75% ethanol [38].

After washing in phosphate buffer, the samples were fixed with 4% (w/v) formaldehyde prepared freshly from paraformaldehyde powder (Sigma), dehydrated by ethanol, cleared with Roti ®-Histol (Roth, Karlsruhe, Germany) and embedded in Paraplast (Sigma), sectioned, and after dewaxing with Roti ®-Histol, samples were embedded in Entellan ® for microscopic examination (Merck, Darmstadt, Germany).

### Transient expression of *CsVDE*-GFP in cucumber protoplasts

To observe the subcellular localization of *CsVDE*, the open reading frame of its cDNA was PCR amplified and fused to the upstream portion of enhanced GFP between the SmaI (5′ end)/BamHI (3′ end) sites in pEZS-NL vector (http://deepgreen.stanford.edu). Because cucumber cotyledon protoplasts were prone to be extracted and transfected [39], we used this tissue as the transient expression material. The procedure of protoplast extraction and transient expression was conducted according to Huang et al. (2013) [39]. Briefly, cucumber cotyledons were cut into fine slices (∼1 mm) and transferred to enzymolysis solution, permeated in a vacuum for 30 min, and then incubated in darkness with rotation (40–50 r/min). The enzymolysate was diluted and filtered through nylon membrane (200 micron mesh). The filtrate was centrifuged and resuspended, then placed in an ice bath for 30 min. 20 µl plasmid (1000 ng/µl), 100 µl protoplasts (2×10^5^ protoplasts), and 120 µl PEG4000-Ca^2+^ solution were combined and mixed gently. The transfection was stopped by diluting the mixture. GFP fluorescent signals were examined with a confocal laser-scanning microscope (Zeiss LSM510 META) in the 488 nm excitation wavelength.

### Immunogold labeling

Specimen preparation and immunogold labeling were conducted essentially according to Zhang et al. (2001) [35]. Briefly, the ultrathin sections were initially incubated with rabbit antiserum directed against CsVDE and then with secondary antibody (goat anti-rabbit IgG antibody conjugated with 10 nm gold, Sigma-Aldrich). The sections were finally double-stained with uranyl acetate-lead citrate and examined with a JEM-100S electron microscope.

### Chlorophyll fluorescence measurements

Chlorophyll fluorescence induction was measured by a PAM-2100 portable fluorometer (Walz, Germany) equipped with a leaf holder. Leaves were dark-adapted for 14 h and exposed to actinic irradiation (1200 µmol m^−2^ s^−1^ PFD) for 0, 0.5, 1, 4, 6, 8 and 10 h. High light was provided by a 400 W metal halide lamp. *F*v*/F*m was measured after 20 min dark-adaptation at room temperature with a leaf clamp.

### Pigment analysis

Leaf samples were taken after high light treatment and immediately frozen in liquid nitrogen. Pigments in the leaves were extracted using ice-cold 100% acetone. The pigment extracts were filtered through a 0.45 mm membrane filter and analyzed with HPLC.

### Fluorescence Video Imaging

Chlorophyll fluorescence was measured at room temperature from fully expanded rosette leaves using a Fluor Cam fluorometer (FC 1000-H). A laboratory-built pneumatic shutter system was used to provide 800 ms saturating light pulses (2000 µmol m^−2^ s^−1^ PFD) during a 1-min illumination with actinic light (500 µmol m^−2^ s^−1^ PFD). The opening and closure of the shutters were controlled by a laboratory-built digital interface connected to a standard personal computer. Color video images of *F*m (maximum fluorescence in the dark-adapted state) or *F*m' (maximum fluorescence in any light-adapted state) were captured during saturating pulses, and false-color images of nonphotochemical quenching (NPQ) were generated as described previously [40].
